# Contestation, negotiation, and experimentation: The liminality of land administration platforms in Kenya

**DOI:** 10.1177/02637758241254943

**Published:** 2024-05-21

**Authors:** Fenna Imara Hoefsloot, Catherine Gateri

**Affiliations:** University College London, UK; Kenyatta University, Kenya

**Keywords:** Land administration, platforms, liminal, Kenya, digitalisation

## Abstract

This paper examines diverse infrastructural interventions in the making of Ardhisasa, the Kenyan state’s digital land information management platform, as a space of contestation, negotiation, and experimentation. We analyse the platformisation of governance through theories on liminality to explain the agency of various actors in shaping the digital state. We particularly zoom into the influence of two actors: the private actors in the land sector and the civil society organisations representing informalised residents, and how they exercise agency in the development of Ardhisasa. Drawing on interviews with state and non-state actors, secondary literature, and extensive experience within Kenya’s land administration system, we trace the overt and covert exercise of power in the platformisation of land administration of Nairobi. Our central thesis is that, despite its progressive development, Ardhisasa follows the tradition of a long line of large-scale infrastructural or developmental projects that rarely deliver on their promise for improvement but rather further entrench marginalised groups due to its exclusion of the already existing, albeit informalised, land administration and transaction practices that meet the needs of the urban poor. We argue that Ardhisasa’s perpetual state of becoming leads to the spatialisation of liminality itself.

## Introduction: Ardhi Sasa, Ardhi Tasa

**Figure 1.**
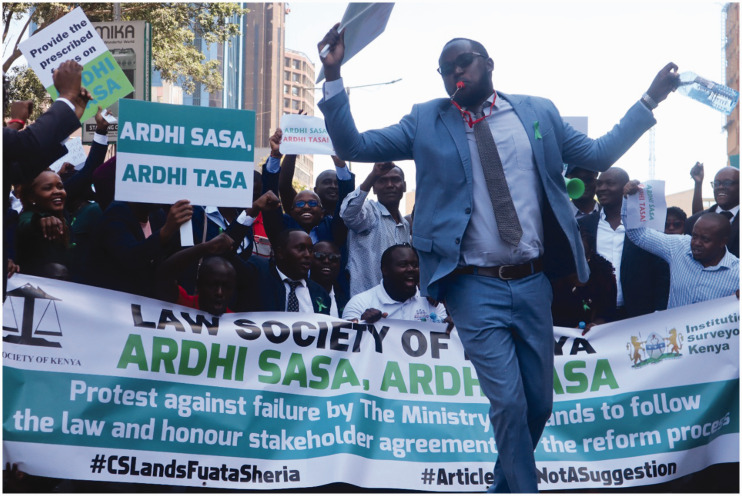
Photo taken during the Ardhi Sasa, Ardhi Tasa demonstration led by the Law Society of Kenya and the Institute of Surveyors of Kenya on 20 January 2023. Image source://twitter.com/lsk_nbi/status/1616450160699097089.

“*Ardhi Sasa, Ardhi Tasa*”. This was written on the banners of the lawyers protesting in front of the Kenyan Supreme Court and the State Department of Lands in January 2023 (Figure 1). It roughly translates to ‘the land now is barren land’, a pun on the name of the national land information management system (LIMS) currently being piloted in Nairobi, known as Ardhisasa. Ardhisasa, a digital land administration platform, is a replacement for earlier paper-based land management in Kenya, which has existed since the colonial period. In line with other digital governance initiatives across the urban spaces, which claim to introduce efficiency, effectiveness, accountability, and the democratisation of public service provision by reducing the frictions associated with transmitting and storing information (Datta, 2018; Graham et al., 2015), Ardhisasa is developed with the promise to reduce the backlog in the processing of land transactions by fast-tracking land property searches, registration, valuation, and issuance of titles, culminating in accelerated investment and development of land as capital. In the longer term, this should resolve land administration and management challenges of manual, paper-based transactions, introduce a more efficient and integrated land management system, and provide a tool to counter fraud and corruption within the land sector. However, the challenges with Ardhisasa’s implementation have delayed transactions, reducing land-based revenues and rendering the land sector unproductive – or barren – at least for the private actors that profit from each trade.

The Law Society of Kenya is only one of the many professional bodies that have expressed discontent with the newly digitalised LIMS and its current design and functioning. Other private sector actors, such as the Institution of Surveyors of Kenya (ISK) representing land surveyors, land valuers, property managers, and land administration managers, have directed complaints to the Ministry of Lands and Physical Planning regarding their day-to-day interaction with the Ardhisasa system and its perceived failure to properly engage with the needs and recommendations of the professionals who will use and depend on this system. While the digital LIMS has generally been welcomed, both the lawyers and built environment professionals argued that the implementation of Ardhisasa has led to the partial failure of services across the land registries and severe delays in land transaction processing. The implementation of Ardhisasa, an ambitious national digitisation program of all land records to replace the manual paper-based system, proves to be one of the most complex and expansive reforms of Kenya’s LIMS to date. It should be understood within a context characterised by bureaucratic lethargy, corruption, and political patronage (Kariuki et al., 2018; Manji, 2020), in which the digitalisation of public services itself is a field of contestation and negotiation.

This paper analyses the platformisation of urban governance (Van Dijck, 2021) through theories on liminality (Elbanna and Idowu, 2022; Mertens, 2018) to explain the agency of various actors in shaping the digital state. In doing so, it responds to calls to forge connections between these fields through the extended governance–digitalization–urbanisation nexus (Barns, 2018; Leszczynski, 2020).

Other empirically grounded studies have analysed digitalisation in urban contexts, either focusing on the reformation of the governance practices (Pelizza, 2017; Taylor and Richter, 2017) or looking at the effects of digitalisation on the position of citizens within the city (Calzada, 2018; Vanolo, 2016). However, little research exists on how, particularly in Southern cities, infrastructures of the digital age generate differentiated urban landscapes through the territorialisation of information systems (Datta, 2023a; Hoefsloot et al., 2022).

To unpack these dynamics, we draw attention to three distinct strategies mobilised by various actors – contestation, negotiation, and experimentation – and are interested in how the three different strategies are leveraged to reconfigure how the digital platform materialises. Our central thesis is that despite Ardhisasa’s progressive development, it follows the tradition of a long line of large-scale infrastructural or developmental projects that rarely deliver on their promise for improvement but rather further entrench marginalised groups (Anand et al., 2018; Lesutis, 2022a; Li, 2007) due to its exclusion of the already existing, albeit informalised, land administration and transaction practices that meet the needs of the urban poor. We argue that Ardhisasa’s perpetual state of becoming leads to the spatialisation of liminality itself by keeping informalised communities permanently on the threshold of becoming formalised conform the digital land administration system.

## The liminality of platform infrastructure

Increasingly, digital platforms such as Ardhisasa are being implemented to govern state services. Previous literature on platforms and platformisation has focussed on the role of big-tech companies in developing digital platforms and their extraordinary power to influence democratic governance (O’Reilly, 2011; Van Dijck, 2021) and the organisation of public, work, and social life (Artioli, 2018; Heeks et al., 2021). We focus on the platformisation of government itself. Platformisation, following van Dijck (2021) and Poell et al. (2019), in this case, refers to the corporate or state-controlled digital information infrastructures that have become central to collecting data, governing the access and circulation of information, and structuring the interaction between users. Digital platforms are often presented as instrumental to knowing the state and guiding daily operations (Kitchin et al., 2016). Yet, they regularly emerge from economic-driven interests and are at odds with pre-existing practices and regulatory traditions (Poell et al., 2019) or reproduce structural inequalities because they are designed for and appropriated by the elites to serve their benefit (Senshaw and Twinomurinzi, 2022). Tracing the development of Ardhisasa as a land administration platform enables us to discern its logic; how the information infrastructure developed is an enactment of the socio-political decisions made and works towards institutionalising these into space and society (Bowker and Star, 2000; Turner, 1967). Highlighting the process, we argue that platformisation will always be an aspiration. It is the internal negotiations and politics which make it interesting and give insight into the emergence of the digital state in all its inequalities (Datta, 2023a).

Guma (2020) states that incompleteness is a common state of infrastructure in Kenya. Building on the work of Simone (2004), he introduces incompleteness as a lens through which to think about infrastructures in the Southern cities beyond normative binaries such as success or failure but rather understanding them as emergent and heterogeneous. Incompleteness, in Guma’s (2020) use of the word, does not fixate on what is missing but turns our attention towards the constructs that are already there and the process of becoming. State infrastructures are often envisioned as technologies for ordering, stabilising, and governing societies. However, in practice, they are the fragmented and open-ended outcomes of the dynamics between the state and society, embodying plural rationales that are not always compatible with the logic of the bureaucratic state (Simone, 2004).

While the concept of incompleteness provides a useful frame to understand the incremental, heterogenous infrastructural development through a non-normative lens (Guma, 2020, 2022), in this paper, we aim to capture the unsettled, ambiguous conditions arising when novel infrastructures implemented create a rupture with previous systems. To conceptualise this ‘interstructural’ (Turner, 1967) moment of movement and transition, of being on the precipice of a new system, we turn to the literature on liminality. Here, it is not the incomplete infrastructure that is the unit of analysis but the liminal space created through this incompleteness that we study. As we will discuss in the next section, where incompleteness focuses on a certain lack of services, liminality emphasises the agency to manoeuvre within the system.

Originating from anthropology to understand rites of passage (Turner, 1967), the concept of liminality, signifying a state of in-betweenness or an ambiguous boundary or threshold, has been applied in geography, innovation sciences, and organisational studies to diverse topics ranging from the iterative process of technological innovation (Mertens, 2018), the position of migrants as belonging to two different places at once (Lim et al., 2016), the strategies of crowd workers within digital platforms (Elbanna and Idowu, 2022), or the transition of work practice during the Covid-19 pandemic (Orlikowski and Scott, 2021), but in all cases it is used to convey the time/space characterised by the possibility for new configurations of ideas and relations to emerge. Kelly and McAdam (2022: 5) define liminality as:

*a transformational space in which individuals may explore possibilities of reconstructing identity and agency. Liminality opens up spaces for tactics including adapting, negotiating, avoiding, rejecting, and resisting, teflonic manoeuvring, legitimacy affirming or contesting, and experimentation, reflection, and recognition.*

Lancione and Simone (2021) similarly argue that liminality is not only a state or space/time but a praxis. Although referring to a state of transition in which the start and end are marked by rituals (Banfield, 2022), in practice, when there is no certainty over the outcome, the liminal period can be suspended to the point where it becomes a semi-permanent condition (Elbanna and Idowu, 2022). Hence, while liminality often invokes a notion of temporality and contains the anticipation of transformation, it might be experienced as perpetual in-betweenness. Liminality becomes the permanent condition of the platform.

Applied to the city, liminality has been used to understand the different modes of urbanisation in the face of uncertainties and ambiguities in urban development policies, political regimes, and market reforms (Müller and Trubina, 2020). Müller and Trubina (2020) conceptualise urban liminality as the space where the rigid infrastructures and rationales of government meet the city's unstable, unanticipated, and unruled assemblages. Highlighting the openings that are created through half-baked laws or semi-implemented plans, scholars have described how actors, from slum dwellers to elites to multi-lateral organisations (Lancione and Simone, 2021; McConnell, 2017; Müller and Trubina, 2020), speculate, improvise, and experiment with codes, spaces, and politics as a process of ‘worlding’, referring to the practices through which the space itself is staged and performed (Omura et al., 2019). The never-ending accumulation of ideas, interventions, and materialities keeps the city on a precipice of whatever it is amassing into (Lancione and Simone, 2021).

This is particularly prevalent in the introduction and use of digital technologies for urban governance. Kitchin et al. (2016) present an understanding of urban digital technologies such as platforms, dashboards, and central control rooms as ‘ontogenic in nature’, centring the different stages of becoming and growth these socio-technical systems undergo before reaching maturity. Whether or not a digital urban technology reaches this point of maturity and passes through the phase in-between design and functioning is dependent on the alignment of state, private sector, and societal actors, technology, and knowledge through the reiterative processes of visioning, negotiation, contestation, and re-visioning (Kitchin et al., 2016, 2017). As we will explain, while Ardhisasa promises a radical transition in Kenya’s land administration system, this is thus far not accomplished, elongating the liminal status of land and digitalisation.

## Agency in in-between spaces

Thinking through liminality allows us to investigate how actors draw on their different capacities to transform the systems they inhabit and how difficulties and contradictions are resolved in practice. Moreover, liminality provides a way to examine how the status of in-betweenness in the city is reproduced in the digital platform by considering the positions that different groups occupy in developing Ardhisasa. This departs from the notion that “*agency is always and simultaneously in-between spaces, positions, and worlds*” (Siles et al., 2023: 64). Siles et al. (2023) operationalise this by analysing how agency in in-between – liminal – spaces is present in the potential and power to act and enact change: to participate in shaping digital technologies through both resistance and compliance.

Understood this way, becoming attuned to the different openings for reconfiguration helps identify the strategies mobilised by actors to exercise their agency and give shape to the platform as an emerging system (Orlikowski and Scott, 2021). Exploring agency within liminal space and time can inform analysis of the platform-user relationships and provide insight into how individuals and organisations mobilise to negotiate, comply, or resist the system's architecture. Within liminality, the ‘rigid hierarchy’ (Jordan, 2015) defining how information systems are organised is mendable, and actors are less bound by the pre-existing identities and roles. Instead, there is the opportunity for individuals and organisations to craft out a new position for themselves within the socio-material system, enact new compositions between state and technology, and derive greater capabilities and outputs. However, this is not a level-playing field. Cultural, context, and regulatory frameworks impact actors’ agency in shaping technological platforms for the city (Odendaal, 2023). Similarly, Elbanna and Idowu (2022) argue that while the theory on liminality foregrounds agency, we should be attuned to the restrictions of culture, society, and politics in structuring and limiting the capacity of people to act within the system. Liminality can be a space/time of opportunity or characterised by vulnerability and loss (Lim et al., 2016).

Hence, we analyse the implementation of Ardhisasa as a liminal space/time of negotiation, contestation and experimentation. While this negotiation happens at many levels and at many times, we particularly zoom into the influence of two actors: the private sector actors, including land surveyors, lawyers, and land valuers, and the civil society organisations representing informalised residents and how they exercise agency in the development of this national LIMS. Considering these two actors and their influence in the design and development of Ardhisasa, we trace the overt and covert exercise of power in the platformisation of land administration in Nairobi. In focussing on these perspectives, we are inspired by Qadri and D’Ignazio (2022), who argue for examining the tactics and agency from within and outside to understand how the platform is built, operated, and resisted.

By positioning the strategies of private sector actors in relation to those of civil society organisations, we follow Côté-Roy and Moser (2019) in their distinction between ‘elite stakeholders’ (e.g. the state and governments, multinational corporations, private foundations and non-profit organisations, and global consultant firms) as holding key roles in the travel of policy and the mobilisation of knowledge, and subaltern actors whose voice, knowledge, and criticism are often actively rejected. They add that with the emergence of foreign direct investment funds and private sector-driven design and building of new urban developments, it is increasingly difficult to distinguish between the state and private actors who form part of the elite (Côté-Roy and Moser, 2019). Kitchin et al. (2017) describe this as the formation of ‘advocacy coalitions’. During the development of digital governance strategies, coalitions between the technocratic bureaucrats and a plurality of consultants, institutional bodies, academics, and civil society organisations are formed and reconfigured that share a particular vision regarding policy and practice and depart from similar values (Kitchin et al., 2017). The formation of the coalition is the negotiation over who is a valuable stakeholder and what politics and imaginaries of the state become embedded. Initiatives that go against the grain of the dominant narrative within the coalition and challenge the state's rationale are often met with apathy or resistance from the inside. Yet, as Li (2007) describes, critical mobilisation can come in many forms and use many strategies for social, structural, and political transformation, from academics using method and writing to the many citizens or recipients of the policies that defy or challenge politics through practice.

Acknowledging how agency is nested within historical, political, economic, and cultural contexts of colonialism, inequality, and informalisation, it is worthwhile to understand these struggles to influence the development trajectory of the Ardhisasa. Specifically, in the context of land administration, we should pay attention to how the digital recording of land is territorialised unevenly. As Cowan (2021) shows, this often happens through facilitating the commodification of land and the strategies of elite state and private sector actors to capture land against the attempts of the urban poor to consolidate their property claims. Lesutis (2022b) describes this as governance through disavowal, or the way in which the state effectively pays lip service to marginalised groups by including them in the discourse and planning of infrastructural developments while rendering them politically absent and materially unaccounted for. As a result, Lutzoni (2016) explains, informality in Kenya is not outside of planning practices but rather emerges as out of a relational sphere of legality, approval, negotiation, as well as contestation, eviction, and delegitimisation.

As we will illustrate in our analysis of the strategies of experimentation, there are many instances where marginalised groups contest their institutionalised exclusion, find ways to manoeuvre within the structure and adapt it to meet their needs, and imagine alternative futures in the process (Kimari and Ernstson, 2020). Kimari and Ernstson (2020) write, “*rather than seeing the innovative infrastructural and incremental practices of the marginalised as making do, or filling the gaps, we can see them as crucial sites to re-think spatial distributions of power*” (p. 9). Creating new forms of information and new ways to record land and property falls within these modes of living between defiance and compliance (Easterling, 2016).

## Methodology

This paper draws on 22 semi-structured interviews and observational data collected during two two-week field visits in 2022 and 2023 within the context of the Regional Futures research project, analysing the process of digitalisation-as-urbanisation in the metropolitan areas of Nairobi, Guadalajara, and Mumbai. We interviewed policymakers and employees from the land administration departments at national and county governments, land surveyors, land valuers, and community organisations representing informalised residents in Nairobi.

The diversity of the researchers being Kenyan and UK-based and our different positionalities in the field provided interesting perspectives. Particularly, we derive information from the experiences of one of the authors who has interacted with Ardhisasa in a professional capacity. Given that Ardhisasa is an ongoing project that has generated a lot of debate in the land sector, being part of the professional bodies working with the platform on a day-to-day basis gave an insider perspective into the practices and debates revolving around Ardhisasa’s development. Moreover, the Kenyan researcher accepted and understood the digitisation process as a land management and administration tool from a historical perspective, having experienced the various previous land reforms in Kenya. In contrast, the UK-based researcher, with no prior experience in the Kenyan land sector, probed and interrogated the land digitisation process from a different perspective, questioning the roles of the different actors in the development of the platform. Additionally, the interviewees showed patience with the UK-based researcher; they were willing to provide detailed responses about the process, whereas the Kenyan researcher was deemed to be knowledgeable about what had been going on and thus received shorter responses.

Interviews were conducted in English and transcribed and analysed thematically, focussing on the digitalisation of land records at the national government, the different actors involved in the development of Ardhisasa, and the challenges that arise due to the implementation of the national LIMS in a context of extensive informality in land tenure. In addition, we attended online and in-person workshops and working sessions organised by professional bodies, such as the ISK and civil society organisations in which Kenya’s land administration and the development of Ardhisasa were discussed. Secondary data from digital media and publications from professional bodies provided more information regarding the public discussions revolving around Ardhisasa’s development and implementation.

## Ardhisasa’s perpetual liminality

Ardhisasa, as a digital LIMS, was initially envisioned as a one-stop-shop designed to enable the States Department for Lands and Physical Planning to modernise the land administration system by improving efficiency and transparency in land transactions through a web-based platform (Kabubu and Wambui, 2021). Kenya’s significant surge in data volumes and rapid population growth have led to notable repercussions on the efficiency and effectiveness of service delivery. The reliance on manual LIMSs within government offices has resulted in long queues and created an environment vulnerable to corruption (Kariuki et al., 2018). Ardhisasa aims to address these challenges by introducing a digital system that promises improved efficiency in land transactions and reduces corruption within the land market.

However, as we will explain in this section, this is a promise that neither Ardhisasa nor its predecessors have completed. Ardhisasa is at best seen as incomplete, a work in progress, or, as described by a land valuer interviewed: “*an ongoing conversation between, uh, the institution of surveyors in Kenya, who will present the affairs of the surveyors and state agents*” (NA221110PV).

This ongoing conversation regarding the development of Ardhisasa has to be understood within a continuous cycle of innovation, incomplete implementation, and reform that has characterised the attempts to digitalise Kenya’s LIMS, starting with the first National Land Information Management System (NLIMS) as part of the state’s e-Government strategy introduced in 2004. Following this first attempt to implement a digital NLIMS, several others have followed (Table 1), each providing the incomplete, sometimes partially discontinued, foundation for the next iteration.

**Table 1.** Timeline of government-led initiatives to digitalise Kenya’s land administration.

Year	Name	Description	Reason for succession
2004	e-Government	Mile-stone initiative to digitalize Kenya’s government transactions, including the Ministry of Lands and Physical Planning.	Continues to form the guiding strategy. After its adoption, several attempts have been made to digitize the land records culminating in the current National Land Management Information System.
2009	First Project on Land Administration in Kenya (PILAK I)	Funded by the Swedish International Development Agency with the objective of improving land administration as part of development of NLIMS to develop business and IT infrastructure, modernize the geodetic framework, reform parcel identification and element of land rent collection system.	Only partially implemented due to continued challenges related to the lack of security of paper records, parcel identification, land rent collection, geodetic reference framework, systematic conversion of Registered Land Act (RLA) titles, staff capacity and awareness.
2013	Second Project on Land Administration in Kenya (PILAK II)	Address the challenges of PILAK I and develop and implement a pilot Integrated Geographic Information System based National Land Information Management System.	The system was to be implemented and funded through MTEF budget for the period 2013–2017 but failed due to lack of funds. Additionally, the added GIS component did not work. Unlike the PILAK I which was donor funded, PILAK II was to be government funded.
2014	Electronic Document Management System (EDMS)	Return to PILAK I and the reorganization of the land registries using an electronic document management system.	The system brought about huge backlogs and only dealt with land registries whilst the department of surveys which is the foundation of land administration was left out.
2017	e-Citizen	Aimed at the digitalization of all citizen-government transactions, including the payments of land rents and the statutory governmental charges in the land information management system.	Like the EDMS, e-Citizen was not GIS-based. The Department of Surveys was not incorporated as the system dealt only on the land registry backlogs in transactions due to lack of integration of the various departments functions.
2021	Ardhisasa	An online platform that allows citizens and private sector actors to interact with land information held and processes undertaken by the national government.	*Currently piloted in Nairobi County.*

NLIMS: National Land Information Management System; IT: ▪; MTEF: ▪

Hailed as the most ambitious attempt to digitalise Kenya’s land administration system to date, in conversations with private sector actors in the field of land administration and management, Ardhisasa was continuously described as having great potential in reforming and streamlining the land administration system in Kenya:

*Ardhisasa is supposed to facilitate all the large transactions and do away with manual processing of transactions. So you can search on it, get your land rate upon demand, a surveyor can carry out a subdivision and launch the whole process on that platform and complete from the comfort of our homes. That is the whole idea. We can carry out complex land transactions without really having to interact physically with the Ministry of Lands. It's a very noble project, I must say. Uh, but of course, it has had its own teething challenges. (K10112022-1)*

With only a few months in operation, the digitisation of the paper-based LIMS has come under fire by both public and private entities, raising questions about whether the current challenges are novel with digitisation or are only now visible as exacerbation of already existing issues in our current land systems. Kenya’s land sector is characterised by historical injustices due to colonisation, long-standing political tensions related to land, and the elite capture of resources and institutions (Boone et al., 2019; K’Akumu, 2016). Aiming to address these problems, the period between 2009 and 2016 has been characterised by drastic reforms in the land sector, most significantly through the new constitution enacted in 2010. Hence, the various iterations of digitalising the LIMS take place amid institutional and legislative reforms changing the administrative landscape in Kenya by decentralising land governance from the national state to the counties through the new Lands Act and the National Land Commission (Boone et al., 2019).

Some of the main challenges prevailing within the platform are related to the fact that with the introduction of Ardhisasa, the Ministry of Lands and Physical Planning is simultaneously working on the unification of the land title system across the country into a block system and geo-referencing the governmental maps to create a national land cadastre. While officially separate projects, these are interlinked since the land records can only be included in Ardhisasa after they have been converted and geo-referenced. Delays in both these processes have led to the incomplete inclusion of land records in the system, making it impossible to access data and verify its status for the areas of Nairobi, which have yet to be converted to the block system, leading to a slowdown of land transactions. Also, in areas that have been converted to the block system, the delays in receiving the results of the search at times took longer than through the previous manual system, elongating the land transaction period (Tarus and Wamae, 2022).

Appearing before a senate committee, the former cabinet secretary for the Ministry of Lands and Physical Planning reiterated that the digitisation process of the Central Registry records (which holds documentation regarding the land previously occupied by the white settlers) was to be completed in 2024 (The Star Newspaper, 2022^1^). Nevertheless, the delays in these processes have significantly impacted the efficiency and service provision through Ardhisasa. One of the platform's essential services, the searches for title authentication – often referred to as an ‘official search’ – is not available for certain parcels of land which have not been digitalised yet, leaving out sections of the city. This standard procedure is the starting point in the process of land valuation and is crucial for all land transactions. Those who have used the system to conduct a search say that the system was, at times, unable to generate results. Various private sector actors in the land sector were frustrated with the lack of information flows to establish the authenticity of the title document: establish ownership, size, type of tenure, encumbrances, and the history of the parcel.

## Contestation and negotiation

At the core of these developments are the private sector actors influencing the design and development of Ardhisasa. While the private sector actors we conversed with at times expressed frustration with the current circumstances, there seemed to be a general patience and willingness to collaborate in developing the platform from which they should ultimately benefit. This is not to say that the relationship between the private sector actors and the Ministry of Lands and Physical Planning is always constructive. One land surveyor explained that Ardhisasa had been developed top-down with little regard for the expertise and needs of the professionals whose work practices will change due to its implementation. “*There have been meetings between, uh, our representative body, which is the institution of surveyors in Kenya, and the Minister of Land*” (NA221110PV). However, he continued to explain that aside from these meetings, there had been minimal consultation with private-sector actors in the design phase. A land surveyor explained that there was a willingness to cooperate and negotiate over the platform's features to ensure that its functionality meets the needs and matches the workflow of the land professionals. Yet, in his experience, the ‘developers’, referring to the programmers coding the digital platform, did not take their input seriously and failed to consider the usability of the platform for surveyors in the field:

*The issue is when […] you want to ensure that what I do in the field, I can do on the system, that becomes complex, and it will take some time to be able to get it to work the way it needs to be worked. [.] If we have an open system where the person who is in charge and the person who is developing are willing and ready to listen to what the users are saying, then [.] it becomes easy. But where you have a developer who seems to think that they know, then you have a problem.* (*NA2303141006)*

Regardless of the ambivalence in experiences from officials during the development of Ardhisasa, the transition from development to the piloting phase created new opportunities for professional bodies to influence the platform's development and employ different tactics. In the development phase, the private sector actors could – to more or lesser effect – express their opinions in conventional settings such as stakeholder meetings, advisory taskforces, and policy position papers. In the pilot phase, different professional bodies have reverted to public protests or expressed their dissatisfaction with the platform in the media in an attempt to influence its progression. This has not been without results.

As a platform, Ardhisasa officially grants access to documents and data by issuing verified accounts for private landowners and private sector actors. However, at its initial implementation, Ardhisasa’s architecture did not recognise land valuers as a relevant group alongside land surveyors and lawyers needing a verifiable account and data to the platform. After repeated protests and lobbying from the professional body of land valuers, a separate account option was created for which they could apply. Similarly, after public complaints from the Law Society of Kenya regarding the fact that foreigners cannot register on the platform – one of the requirements for an account is a Kenyan ID number – the Ministry of Lands and Physical Planning has responded that they will work towards making this possible. It is in these interactions that Ardhisasa shows itself as a responsive platform, accommodating the needs and perspectives of private sector actors as key users and stakeholders in the process.

Nonetheless, complaints regarding the changing workflows within the system have not been taken on thus far. In the paper-based LIMS and the digital predecessors of Ardhisasa, anybody with a stake in a piece of property that had been registered was free to ask for and receive an official search in any of the land registries. This enabled people and organisations to get information on the ownership and encumbrances of any registered land parcel whenever they choose, providing they pay the necessary statutory fees. Currently, in a break with custom, Ardhisasa requires the property owner's permission to be sought before conducting official searches. The negotiation about the workflow is, in essence, a discussion regarding the trade-off between transparency in information and the privacy of property owners, which is currently being re-settled in practice and through debate in meetings and publicly on social media and through protest.

In addition to negotiating over the platform’s architecture, Ardhisasa’s introduction has forced private sector actors in the land sector to adapt their practices. Adjusting the socio-material system through developing new relationships or deepening existing ones proves to be crucial to maintain information flows even if the digital platform does not produce the results required. According to a government planner interviewed informally, some land professionals have managed to continue with land transactions because they have a liaison person whom they consult to facilitate the process. Actors who do not have an insider person are forced to interact with a “silent” platform that will not lead fast results:

*The frustrating bit is that you don't know who to call. Because once you launch it on the platform, it's just wait and wait and wait unless you have an inside contact, and I have no idea what they're doing now on the inside. I'm not sure if they have access to the system and can see you applied for the search. I have no idea. You have to pay someone inside to do it for you, because you can't proceed with the valuation unless you have the searches. This also applies to the bankers and the lawyers who are dependent on the search document to affect any transaction. (K10112022-1)*

Taken together, these expressions of frustration, experiences with transformation, and the need for adaptation from the land professionals are illustrative of the liminal stage of Kenya’s land administration system. It is in between paper and digital-based systems, in between the development and implementation phase, and in between previous innovations and future ones. As described earlier, the constant reinvention of digital platforms for land administration causes a seemingly never-ending transitional phase where one digital platform serves as a starting point for the next iteration, each different from the previous one through incremental innovations. However, none has been implemented fully and embedded in land administration practices.

Hence, while these forms of resistance and cooperation could be interpreted as opposition or disapproval for Ardhisasa, these are also spaces for negotiation and improvement. It is through these series of *ad hoc* interactions and conversations, some conducted in formal settings while others happen behind closed doors or on the street, that Ardhisasa is taking shape. In this proactive form of engagement with the development of the digital platform, actors also find ways to overcome the precarious position they hold and build more secure positions within the system that align with their previous roles in the paper-based system. For Ardhisasa, this means that although clear decisions on how to impact the development trajectory of the platform can be made, rules for interaction can also be withdrawn, adjusted, or adapted.

## Experimentation

Outside of the core of ‘elite stakeholders’ (Côté-Roy and Moser, 2019) such as the private sector actors, civil society is peripheralised in Ardhisasa’s development. One of the main concerns with the introduction of Ardhisasa has been the issue of digital illiteracy and inclusion. In Kenya, 17% of the adult population has low illiteracy levels and an even higher amount of people, the majority women, are not fully digitally literate or only have limited access to digital technologies such as computers, smartphones and the internet (Koyama et al., 2021; UNESCO Institute for Statistics (UIS), 2022). Moreover, while Swahili is the more commonly spoken language in Kenya, currently, the platform is only provided in English. As a result, a large part of the Kenyan population will not have the full capacity to operate Ardhisasa in its current form. This has raised calls to maintain a paper-based system to fall back on in addition to the digital platform:

*So, we need to be inclusive […] and we have to look for another alternative, which gets us back to, takes us back to paper. […] If you do not consider paper and then you end up leaving a section of people away. […] The government is digitising land records, and that means you're going to digitise your land rights.* (NA22110CS) 

Nevertheless, what is less discussed is the effect of digitalisation on those for whom the paper bureaucracy also does not function. Spread across Nairobi are informalised settlements that house around 60 per cent of the urban population, assembled and expanded through unregularised land transactions and auto-construction. Like so many other informalised settlements around the world, these neighbourhoods always seem to be many things at once. They are icons of the resilience and self-sufficiency of urban residents but also in need of upgrading and development; they are examples of pioneering and bottom-up urbanism but also need state regulation and service provision; they are the steppingstone into the city while also being the embodiment of exclusion and marginalisation; they are vehicles for incremental consolidation while also being perpetually unfinished.

Truelove (2021) describes the ability of informalised settlements to represent so many things while being in between everything as liminal infrastructural space. These spaces are neither regularised nor sanctioned, legal nor illegal. More importantly, they are in constant transformation. They are spaces of continuous struggle to transition into more formalised urban assemblages, of which a crucial step is the regularisation of land titles and urban residency. The introduction of Ardhisasa means that land possession has to be registered on paper and in the digital LIMS. A lack of official landholding documents is automatically associated with non-ownership.

Yet, parallel to the formalised land title registration system, many other forms of land governance and transfers exist within Nairobi, which Ardhisasa does not cater to. In Nairobi’s land market, a variety of alternative documents – share certificates, temporary occupation licenses, allotment letters, certificates of tenure, and subleases – are used to transfer land as proof of ownership in informal or semi-formal ways. While the state does not recognise these documents as confirmation of land ownership, they are generally accepted in property and land transactions as documenting unregistered housing and proof of continued occupancy. Hence, they are intermediary documents that play an important role in transitioning from informalised to formalised residency and ownership or, for example, determining who should be included in slum upgrading schemes and resettlement programmes. However, as they only establish a legitimate interest in the land rather than ownership, and are often not issued by a formal authority but are drafted amongst community members, they cannot be registered within Ardhisasa.

Lacking documentation is not only an issue with people living on squatted public or private land. Partly due to the backlog in registrations and the resulting lengthy bureaucratic process, many people do not follow through with registering transactions as a result of succession, inheritance, or sub-divisions. As these transactions are often only processed informally without officially changing the land title, disputes over land ownership and land use claims in Nairobi are prevalent. With the digitalisation of land records, property owners face a lack of administration and formal paper-based and digital registration of land titles.

Multiple community-led groups and civil society organisations in Nairobi and Kenya at large have aimed to raise issues related to digital inclusion and the registration of people living in different degrees of informality through various means, such as lobbying, organising multi-stakeholder workshops, and solicited and unsolicited advice. Most notably, there have been initiatives to take means into their own hands to map and register irregular settlements by and for the community. They use various digital and in-person means to do this, overcoming the constraints of the existing digital platforms. As a civil society organiser stated: “*Government has been absent for a long period of time. And when government is absent, then people innovate*” (NA230317I011).

In this case, the innovation she was referring to was the introduction of an alternative land registration system – the social tenure domain model (STDM) – that departs from the relationship between an individual and the space that they occupy rather than the documents they possess. The STDM functions as a tool for land administration that breaks down the complexities that exist within irregular settlements. It allows us to identify the communities and individuals living on squatted land and define and establish their relationship with space and role within the land market. For example, individuals can be registered as tenants, structure owners, or landlords. Moreover, exploring the opportunities and boundaries provided by the Community Land Act, the STDM facilitates the registration of communal lands in informalised urban neighbourhoods (Odol Otieno et al., 2017). While the Community Land Act was introduced in 2016 to formalise rural community lands, it provides a comprehensive definition of community, opening pathways for urban collectives living on the same land, sharing customs, culture, or language, to pursue the registration of the territory they occupy as community land.

The primary motivation for introducing the STDM in Nairobi is to expand the categories of formal categories of land tenure to include the relationships with land and occupancy that have emerged in informality. This challenges the structures determining ‘legitimate’ land tenure and works towards decriminalising the auto-construction of housing and basic services such as water and electricity in informalised settlements, hoping to reduce forced evictions and provide tenure security.

On a more profound level, the experiments with community mapping, the STDM, and registering irregular urban settlements under the Community Land Act challenge the assumptions in the architecture of Ardhisasa that value private ownership and public administration structures over communal ownership or collective land administration and management. Where private sector actors such as land valuers, lawyers, and surveyors are making progress in influencing Ardhisasa to become a faster, more accessible, and more efficient platform, the community organisations pushing these alternatives want something far more radical. They want to introduce a non-capitalist land administration system that prioritises communal over private ownership and appreciates the African sociocultural dimensions of land. In discussing the tensions between the formal systems of the state and the informal and semi-formal documentation of communities that want to become formalised, a community organiser explained the challenges the assumptions built into the NLIMS:

*So how do we legitimise their innovations to form a basis of policy development, for instance, in the land sector? There are layers of rights that people have in the informal settlements. They are user rights. They are access rights. They are occupancy rights. […] That has been the contestation between the formal Kenya and the informal Kenya that forms the majority. And it will lead us to our conversation in the National Land Information Management System. […] The National Land Information Management System, as it was built, does not appreciate the people's interpretation and the cultural interpretation of land as property in the African context.* (NA230317I011)

The liminality of the land administration system – a time/space and praxis in which the categories are still malleable – allows for these types of innovations, tensions, and discussions to emerge. It provides an entry point for civil society organisations to introduce alternative approaches that question the basic premises of the platform. Yet, we notice that while the private sector actors have exerted significant pressure on the development of Ardhisasa as a platform, civil society organisations and community representatives have had little real influence to date. Ardhisasa is constructed through politics, knowledge, and actors and emerges from a market-driven governance approach. The objectives of the land professionals gel well within the neoliberal imaginary of the digital state that Kenya is trying to establish.

On the contrary, community groups and civil society organisations propose a radical alternative that challenges the platform's core objectives and is therefore not included in the conversation. This illustrates the idle attempts of community groups and civil society organisations to be recognised as important actors in the digitalisation of land records. As a result, people living in various degrees of liminal infrastructure space in Nairobi continue to be blank spots in the platform. They operate from the margins, socio-spatially distant and outside of the information systems governing land.

## Conclusion: Spatializing liminality

Looking critically at the negotiation over the platform and whose voice is represented or not in the digital land administration system can inform us how notions about legitimate tenure, ownership, and land governance are embedded in the platform's architecture. In this paper, we specifically did not focus on the formal institutions and how they have built Ardhisasa. Instead, we analyse the platform’s emergence through the conversations, contributions, and conflicts with the private sector and societal actors that Kenya’s land administration relies on as a liminal time/space and praxis.

Liminality is crucial in three ways for how we conceptualise the platformisation of the land administration system. First, liminality evokes the history of digitalisation in the land sector and places Ardhisasa in relation to the previous platforms developed over time. Over the past two decades, there have been ongoing initiatives to digitise land records and subsequently create a digital platform for Kenya's national LIMS. However, the result that private sector actors and citizens experience right now is far from what was intended. The various cycles of design, implementation, and abandonment of policies and digitalisation efforts have turned what was promised to be an all-encompassing e-Government strategy into an enduring process of contestation, negotiation, and experimentation, which never really seems to reach completion.

This is described by Kitchin et al. (2017) as the ‘last mile’ problem, referring to the fact that many e-governance policies and digital technologies for the state, such as platforms and urban dashboards, never seem to reach maturity but remain in ‘pilot’, ‘testbed’, ‘experimental’ or ‘living lab’ stages. Nevertheless, it is not just an issue of incompletion. It is an issue of struggle and frustration. Whilst Ardhisasa, like many other state-led projects in Kenya, tries to present a smooth transition and a coherent set of practices for land information management and service delivery, reality proves to be more precarious (Lesutis, 2022a). In a context as complex and fraught as land administration in Kenya, there are multiple objectives and rhetoric that continually bump heads with each other, sometimes through negotiation and experimentation and sometimes through contestation, leading to an open-endedness in the process of platformisation.

Secondly, the lens of liminality highlights how the development of digital platforms is situational, power-laden, and a politically ambiguous praxis. Ardhisasa reflects the very ambiguities that gave rise to it in the first place. In its liminality, Ardhisasa exists in between the ambitions of Kenya’s authorities to reduce bureaucracy and corruption and the social and political imperatives of a highly unequal state and urban society. These often conflicting foundations allowed negotiation over and experimentation with the rationale and function of the platform and its uses.

In our analysis, we note that Ardhisasa is remarkable in its flexibility in incorporating feedback from private sector actors. The digitalisation of public services through diverse infrastructural interventions – e.g., the LIMSs, provides an opportunity for the private sector actors to reconfigure inherently political socio-technical relations and consolidate their position within the system. The platform is expanded or adjusted through negotiations with land professionals without challenging its underlying principles. At the same time, Ardhisasa has proven to be resistant to the efforts of civil society organisations that challenge the assumptions in its architecture and, by extension, the neoliberal politics of the platform. These actors remain in the periphery, outside of the geographical spaces and digital structures of power (Datta, 2023b).

Returning to Siles et al. (2023: 66), the establishment of user-platform relationships through these modes of expressing agency can be seen as a process of worldling (Omura et al., 2019): “*staging the world in ways that render certain realities as neutral*”. The negotiations over the platform’s functions mirror the inequalities present in Kenya’s urban and political landscape. As we found in our analysis, while various actors mobilise their agency to shape Ardhisasa in various ways, their power for influence differs greatly to the extent that it has been normalised that private sector actors are influential actors while civil society is kept at the margins. From this perspective, Ardhisasa is reductive, simplifying complex relationships between people, land, and the state into decontextualised and tidy categories of legitimate and illegitimate ownership and having a legitimate or illegitimate stake in the platform. This mirrors the dynamics described by Kimari and Ernstson (2020), where large-scale infrastructural projects in Kenya reproduce the landscapes of empire and capitalist production under the banner of ‘development’ or ‘modernity’, neglecting citizens’ actions that envision more just, communal, or non-capitalist futures.

Third, Ardhisasa creates liminal space. The development of the LIMS is inherently situated in space and the inequalities existing with the city and land sector. Analysing the development of the Ardhisasa in relation to the experimental alternatives proposed by civil society organisations in Nairobi casts a light on how the platform’s architecture structures the urban landscape in profound ways. The liminality of the digital platform and the fact that it is incomplete in its implementation – not all the data is included – nor in its scope – not all land transactions can become digitalised – contributes to liminal existence in informalised settlements. Hence, considering the relationship between the liminal digital platform and the liminal city, we note the futility of approaching ‘land’ and ‘digital’ as separate fields of urban governance but rather argue for a critical analysis of digital-land relations and the territorialisation of information infrastructures. Informalised communities are permanently on the threshold of becoming formalised, but with the liminality of the land administration system, never seeming to complete this transition.

Hence, through the perpetual liminality of the LIMS implemented in Kenya, liminality itself becomes spatialised (Banfield, 2022). In many ways, this is a silent process of further exclusion of already disenfranchised communities living in conditions of semi-formality and informality by increasing uncertainty. You cannot see it in the data or hear it in the discourse regarding land possession and administration. But it is lived and resisted every day – in the informalised communities, in the land transaction documents that are not recognised, and in the experiments with communal land. As Ardhisasa is currently piloted in Nairobi, we have focussed our analysis on its impact on informalised urban settlements. However, as Ardhisasa completes its anticipated rollout nationally, it will become important to consider the future impact on Kenya’s rural and indigenous communities, which are materially, politically, and informationally underserved by the state’s institutions.

## Note

1 https://www.the-star.co.ke/news/2022-03-03-digitisation-of-land-records-to-be-completed-in-2024–karoney/.
